# Chitin Research Revisited

**DOI:** 10.3390/md8071988

**Published:** 2010-06-28

**Authors:** Feisal Khoushab, Montarop Yamabhai

**Affiliations:** School of Biotechnology, Suranaree University of Technology, Nakhon Ratchasima, 30000, Thailand; E-Mail: fl.khoushab@gmail.com

**Keywords:** chitin, chitosan, chito-oligosaccharide, application, nanotechnology, biotechnology, nanobiotechnology

## Abstract

Two centuries after the discovery of chitin, it is widely accepted that this biopolymer is an important biomaterial in many aspects. Numerous studies on chitin have focused on its biomedical applications. In this review, various aspects of chitin research including sources, structure, biosynthesis, chitinolytic enzyme, chitin binding protein, genetic engineering approach to produce chitin, chitin and evolution, and a wide range of applications in bio- and nanotechnology will be dealt with.

## 1. Introduction

Chitin is one of the most abundant renewable biopolymer on earth that can be obtained as a cheap renewable biopolymer from marine sources [1]. It is biocompatible, biodegradable and bio-absorbable, with antibacterial and wound-healing abilities and low immunogenicity; therefore there have been many reports on its biomedical applications [2]. Accordingly, a very broad range of applications in different fields such as food technology, material science, microbiology, agriculture, wastewater treatment, drug delivery systems, tissue engineering, bionanotechnology have been reported.

Henri Braconnot, a French professor of natural history, discovered chitin in 1811 after the discovery of a “material particularly resistant to usual chemicals” by A. Hachett, an English scientist in 1799, and in 1843 Lassaigne demonstrated the presence of nitrogen in chitin [3]. Henri Braconnot’s name for chitin was fungine. In 1823, Odier found the same material in insects and plants and named it chitine [4]. Since all chitin-based materials are derivatives of chitin, in this paper the word chitin is used generally to describe both chitin and its derivatives unless otherwise mentioned in the text.

## 2. Source

Chitin, a poly-beta-1,4-*N*-acetylglucosamine (GlcNAc), is the main component of arthropod exoskeletons, tendons, and the linings of their respiratory, excretory, and digestive systems [5,6]. It is also found in the reflective material (iridophores) both in the epidermis and the eyes of arthropods and cephalopods (phylum: *Mollusca*) [6]. Chitin is also an important component of the cell wall of fungi [7]. Moreover, there is one report - based on lectin binding, endo-chitinase binding and enzymatic degradation studies - that the epidermal cuticle of a vertebrate named *Paralipophrys trigloides* (fish) is chitinous [8]. Therefore, chitin is not only an essential component of invertebrates but may also be present in vertebrates. Unlike cellulose, chitin can be a source of nitrogen as well as carbon (C:N = 8:1) [9].

## 3. Structure

Chitin contains 6–7% nitrogen and in its deacetylated form, chitosan contains 7–9.5% nitrogen. In chitosan, between 60 to 80% of the acetyl groups available in chitin are removed [10]. There are three forms of chitin: α, β, and γ chitin. The α-form, which is mainly obtained from crab and shrimp shells, is widely distributed. Both α and β chitin/chitosan are commercially available. The α-chitin chains are aligned in anti-parallel fashion. The anti-parallel arrangement in α-chitin gives rise to strong hydrogen bonding and consequently makes it more stable [11]. The β-form mainly obtained from mollusks such as squid, is arranged in parallel, whereas the γ-form contains two parallel and one anti-parallel strands of chitin [12]. Conversion from the β-form to the α-form is possible, but not the reverse [13–15]. γ chitin can be converted to α chitin by treatment with lithium thiocyanate [16].

Chitin is insoluble in water due to its intermolecular hydrogen bonds [17]. But water-soluble chitin-based derivatives such as chitosan or carboxymethyl chitin can be obtained. One of their most important features is the ability (flexibility) to be shaped into different forms such as fibers, hydrogels, beads, sponges, and membranes [18]. The origin of chitin affects its crystallinity, purity, polymer chain arrangement, and dictates its properties [19].

## 4. Chitin Biosynthesis

Chitin biosynthesis has been studied in a large variety of organisms. The enzyme for this synthesis is called chitin synthase (CS). Three CSs have been found in *Saccharomyces cerevisiae* CS I, CS II, and CS III. They are different from each other in terms of function and catalytic activity. Most of chitin is synthesized by CS III [20]. Fungal CS are grouped into two families and five classes [21]. There are two CS genes in insects [22,23]. Nematodes possess two chitin synthase genes, which are differentially expressed [24]. Therefore, there seems to be specialization among chitin synthases in different organisms where different enzymes carry out different functions. CSs use UDP-*N*-acetylglucosamine as substrate to produce chitin fibrils [25]. This was proven in an experiment done on *Mucor rouxii* [26]. The genomes of some chloroviruses contain a chitin synthase gene (*cs*). By introducing the gene of chlorovirus CVK2 into *Chlorella* cells, an algae that does not possess a chitin synthase gene, this microorganism could be engineered to produce chitin [27].

Several enzymes, so called DG, have been isolated from the *Xenopus laevis* embryos that are differentially expressed during the gastrulation stage. One of these enzymes is DG42. It is expressed from midblastula to the end of the neuralation stages. DG42 synthesizes a Nod-like chitin oligosaccharide. Nod is the enzyme responsible for producing chito-oligosaccharide in *Rhizobium* [28]. It seems that while this enzyme is expressed during early embryogenesis the amount of hyaluronic acid (HA) increases. It has been suggested that chitin oligosaccharides are present at the reducing ends of HA chains. Thus, it has been proposed that chitin oligosaccharide produced by this enzyme acts as a primer for synthesizing HA. Homologs of DG42 have been found in zebrafish and mice [29,30]. They also synthesize Nod-like chitin oligosaccharide during early embryogenesis. However, it remains controversial whether DGs are chitin synthase or hyaluronan synthase. Since DG42 has been isolated from frogs, zebrafish and most importantly mice, further study is needed to investigate whether or not this enzyme is widespread throughout mammals including humans, and if DGs is shown to synthesize chitin in animal, the role of chito-oligosaccharide in embryonic development should be further investigated.

## 5. Chitinolytic Enzymes

Chitinases, which hydrolyze chitin, are present in a wide range of organisms including viruses [31], bacteria [32], fungi [33], insects [34], higher plants [35,36], and mammals [37]. Most organisms (bacteria, plants and insects) have large families of chitinases with distinct functions, including digestion, cuticle turnover, and cell differentiation. Based on amino acid sequence similarity they can be grouped into glycosyl hydrolase families (GH) 18 and 19, which are structurally unrelated. The catalytic mechanism of chitinases family 18 involves substrate-assisted catalysis, which retains the anomeric configuration of the product [38]. They are ubiquitous with an (alpha/beta) 8-barrel fold structure in the catalytic domain [39]. Family 19, glycosyl hydrolases, share a homologous catalytic domain. They consist mainly of alpha helices. Their catalytic mechanism is a general acid-base mechanism that inverts the anomeric configuration of the hydrolyzed GlcNAc residue [38]. Family 19 chitinases have mostly been identified in plants [40]. Both GH 18 and 19 chitinases have signal peptides, indicating that the enzymes are secreted and function outside the cells.

Family 18 of glycosyl hydrolases include active chitinases and inactive chitinase-like proteins or chito-lectins, which lack endogenous chitin and have been found widely in mammals [41]. Recent comparative genomic analysis to address the evolutionary history of the GH18 multiprotein family from early eukaryotes to mammals, revealed that the GH18 chitinase involved in an emerging interface of innate and adaptive immunity during early vertebrate history [41]. Chito-lectins are inactive due to lack of some critical residues in their catalytic sites. Several of these chitinase-like proteins have been identified such as oviductin or oviduct-specific glycoprotein [42], YKL-39 [43], HC gp39 and YKL-40 [44–46] in human, YM1 [47], YM2 [48], ECFL [49], breast regression protein 39 (BRP39) [50] in mice, and gp38K [51] in pigs. Their functions are unknown, however, it is speculated that they may be involved in fertilization [52], chemotaxis [49], and tissue remodeling [53]. Besides these inactive chitinases, two active chitinases have been found in humans, these are chitotriosidase [54] and acidic mammalian chitinase (AMCase) [55]. Chitotriosidase can cut both chitin oligomer and colloidal chitin; its optimum pH is broad, and it is mainly secreted from macrophages [56]. AMCase is active in acidic pH, it is mostly expressed in stomach and lung, its gene has 11 exons and is located on chromosome 1 [57]. Interestingly, Glyco_18-containing proteins are established biomarkers for human diseases, and both human chitinases and chitinase-like proteins have been used as indicators of inflammation and cancer [58].

Bacterial chitinases belong to both family GH18 and 19. Most of their structure-function studies came from the studies of the enzymes from *Serratia marcescens* [59–61], *B. circulans* [62–67], and *Vibrio* spp. [68–70].

Recently, chitinases have gained interest in different biotechnological applications due to their ability to degrade chitin in the fungal cell wall and insect exoskeleton, leading to their use as antimicrobial or insecticidal agents. [71–73]. Another interesting application of chitinase is for bioconversion of chitin, a cheap biomaterial, into pharmacological active products, namely *N*-acetylglucosamine and chito-oligosaccharides [74–76]. Production of chitin derivatives with suitable enzymes is more appropriate for sustaining the environment than using chemical reactions [77]. Other interesting applications include the preparation of protoplasts from filamentous fungi [75], bio-control of insects and mosquitoes as well as the production of single cell protein [75,76]. Thus, there have been many reports on cloning, expression and characterization of chitinases from various organisms, including bacteria, fungi, plant and animals [75,78–80].

## 6. Chitin Binding Proteins

Kawabata *et al.* identified a molecule, named tachycitin, from hemocytes of a horseshoe crab with chitin binding activity. This is a small molecule that is 73 residues long and possesses five disulfide bonds [81]. A major protein in the chitin-walled cyst of protozoan *Entamoeba histolytica* also contains chitin binding domains. This protein is a lectin named EiJacob1 [82]. In addition, a chitin binding lectin has been isolated from Stinging Nettle Rhizomes [83]. Several genes encoding proteins with chitin binding domains from arthropods have been identified by whole genome sequence analysis [84]. Two proteins with chitin-binding domains have been identified from silkworm, *Bombyx mori* [85]. In addition, it has been shown that *Tribolium castaneum*, red flour beetle, contains genes encoding proteins peritrophin A-type chitin-binding domains [86].

Chitin binding proteins have also been isolated from plants. They belong to the lectin superfamily and are secreted from the plants as a part of their defense system [87]. Most of plant chitin binding proteins have a conserved motif, called havein domain or chitin binding domain [88].

A chitin binding protein with antifungal activity has been isolated and characterized from *Streptomyces tendae* Tü901. This protein binds to the surface of germinated conidia and tips of growing hyphae, changing the growth polarity of the fungi [89]. Other organisms in which chitin binding proteins have been identified include sweet potato hornworm, *Agrius convolvuli* [90], *Amaranthus caudatus* [91], Ginkgo biloba [92], and *Streptomyces reticuli* [93]. There is a conserved motif, known as RR consensus motif in the arthropods cuticular proteins, which along with its extended forms, are considered as chitin binding motifs [84,94,95].

Various chitinolytic microorganisms have been shown to produce non-hydrolytic accessory proteins that increase enzyme efficiency [96], such as the Gram-negative soil bacterium *Serratia marcescens*, which uses three different family 18 chitinases to degrade chitin. It has been reported that a small non-catalytic protein, CBP21, which binds to the insoluble crystalline substrate, leading to structural changes in the substrate and increased substrate accessibility, is required for efficient chitin degradation [97].

## 7. Genetic Engineering Approach to Produce Chitin

It is difficult to obtain pure carbohydrates, especially chitin, through conventional techniques. Bacterial cells have been engineered in an effort to overcome this problem [98]. *E. coli* has been engineered to produce chitobiose. This method took advantage of NodC, which is a chito-oligosaccharide synthase, and genetically engineered chitinase to make a cell factory with the ability to produce chito-oligosaccharides [99]. Recombinant chito-oligosaccharides have also been obtained using *E. coli* cells which expressed *nodC* or *nodBC* genes [100]. By expressing different combinations of *nod* genes in *E. coli*, *O*-acetylated and sulfated chito-oligosaccharide have been produced [101].

## 8. Chitin and Evolution

There are several reports indicating that chitin plays a role not only in those organisms in which its role is expected, such as in fungi and crustaceans, but also in other organisms. It has been shown that Rhizobia-mediating nitrogen-fixing nodules possess a signal molecule necessary for the root nodulation process termed lipo-chitin oligosaccharide, which comprises a β-1,4-linked *N*-acetylglucosamine (GlcNAc) tetra- or penta-saccharide [28,102]. *N*-acetylglucosamine has been reported to play a role during development of moulds as well [103].

The presence of intracellular and extracellular GlcNAc-containing compounds, in the forms of *N*-glycans and intracellular glycans, indicates the role of chitin and its derivatives from an evolutionary point of view [104]. Finding a role for chitin derivatives in animals was an important discovery. Semino *et al.* (1997) proved the importance of chitin oligosaccharides in the embryogenesis of zebrafish and carp [105]. GlcNAc containing muscle cell surface molecules have been shown to possess the ability to guide neurites through extracellular matrix [106]. It has been reported that the addition of *O*-linked GlcNAc (*O*-GlcNAc) to the proteins in nucleus and cytoplasm is different from classical *O*-glycosylation of the secretory pathways [107]. This reversible post-translational modification [108] modifies serine and threonine residues [109] and seems to play an important role in apoptosis [110], death signaling of mouse embryonic neuroepithelial cell [111], neurodegenerative disorders [112], signal transduction [113,114], synaptic plasticity [115], transcription and translation [116,117], stability of target proteins [118], proteasomal function [119], cancer [120], diabetes [121], subcellular localization [122], and nuclear transport [123]. *O*-GlcNAc is also important in the cardiovascular [124], and immune systems [125]. Considering the fact mentioned in part 4 about the role of chitin synthase and chitin-oligomer in mice, which is close to human species, and that humans have chitinase genes, it would not be surprising if future studies found one or more roles for chitinous materials in humans at some point in human evolution and/or embryonic development.

## 9. Applications

### 9.1. Immunology

The key property of chitin-derived products for application in various biomedical applications is the immuno-modulating effect [126,127]. Various mechanisms of immuno-enhancement activity of chitin and its derivatives have been reported. For example, chitosan exhibited the ability to boost NO production from macrophages in the presence of interferon-γ (IFN-γ) through the NF-κB signaling pathway [128]. In addition, Minami *et al.* (1998) found that chitin and chitosan affected C3 and C5 components of complement system and concluded that complement system is activated by chitin and chitosan through the alternative pathway. After activating the complement, C5a is produced followed by an increase in migration of polymorphonucleate (PMN) to the injured tissue. This is a normal inflammatory reaction but in the presence of chitin and chitosan, there are no inflammatory symptoms, such as erythema, temperature elevation and abscess formation [129]. The intensity of complement [129] and macrophage [130] activation of chitin is less than chitosan; therefore, chitin is more biocompatible.

### 9.2. Hemostasis and Wound Healing

Hemostasis through blood coagulation is an important step for wound healing. The main cellular components in blood coagulation are platelets. It has been shown that chitosan has a hemostatic effect [131]. Okamoto Y. *et al.* reported that chitin is an effective agent for hemostasis maintenance through aggregating platelets, and suggested that the effect of chitin and chitosan is due to both physical and chemical properties of these biopolymers, especially their amino groups [132]. Shelma *et al.* (2008) developed a composite film by using chitosan and chitin. They used chitin nano-fiber to improve the composite’s tensile strength and elasticity [133]. Mi *et al.* designed an asymmetric chitosan membrane with the ability to protect skin by preventing bacterial invasion and halting the evaporation of the skin’s water, two important factors for dressing wounded skin [134,135]. A chitosan acetate bandage has been shown to have good antibacterial activity when applied to burnt skin contaminated with *P. aeruginosa* [136].

### 9.3. Scaffold for the Regeneration of Tissue

Chitin and its derivatives have been used as scaffolds for bone and other natural tissue regeneration [137] as well as structures by which three-dimensional formation of tissues are supported [138]. While looking for a good material for a good scaffold, there are at least four important factors that should be taken into account: (1) ability to form temporary matrix, (2) ability to form porous structure for tissue to grow, (3) biodegradability, and finally (4) non-toxic byproducts from the digestion [139,140]. Thus, neither the physical nor biological properties of such biomaterials should be ignored [137]. Chitin and its derivatives have been shown to possess these criteria.

### 9.4. Neuro-Tubes Guided Nerve Regeneration

Based on the fact that chitin has high mechanical strength under physiological conditions (low for chitosan) chitin has the potential to be a good nerve guidance channel. Ferier *et al.* used this fact and made chitin tubes that could support nerve cell adhesion and neurite outgrowth [130]. In a research related to nerve regeneration, it was shown that rabbits with the crushed common peroneal nerve exhibit better improvement in peripheral nerve regeneration in the presence of chitooligosaccharide. As a result, chito-oligosaccharide can be used as neuroprotective material with an ability to improve injured peripheral nerve regeneration [141].

### 9.5. Blood Cholesterol Control

Chitin and chitosan are among the candidates to battle obesity and hypercholesterolemia. It has been reported that they can reduce the amount of cholesterol in rats [142]. Several mechanisms have been proposed to explain this phenomenon. One is through electrostatic interaction between lipids and aminopolysaccharides [143]. Chitin binds to lipid (cholesterol) micelles and inhibits their absorption. Another proposed mechanism is increasing the excretion of bile acid by which the amount of fecal fat increases [144]. Feeding mice with chitosan showed hypocholesterolemic effects. It seems that the mechanism of chitosan’s cholesterol-lowering effect is through suppression of food intake [145]. The hypocholesterolemic effect of chitosan has also been found in humans. When 3–6 g/day of chitosan was given in the diet to eight healthy males, total serum cholesterol significantly decreased, and when the ingestion was stopped, the value increased to the level before ingestion. The proposed cholesterol lowering mechanism of chitosan was that it combines bile acids in the digestive tract, and excretes them into the feces, thus decreasing the resorption of bile acids, so that the cholesterol pool in the body was decreased and the level of serum cholesterol consequently decreased [146].

### 9.6. Drug Delivery Carriers

It is important for a drug delivery carrier to be efficiently removed after delivering drugs. In other words, it must not accumulate in the body nor must it be toxic [147]. Chitin derivatives such as *N*-succinyl-chitosan [148], carboxymethyl chitin [147], chitosan hydrogel [149], and hydroxyethyl chitin [150] have been shown to possess such characteristics.

Chitosan in the form of colloidal structures can entrap macromolecules by various mechanisms. These associated macromolecules have been shown to transport through mucosa and epithelia more efficiently [151]. Cationic chitosan in combination with other natural polymers has been shown to enhance the drug encapsulation efficiency of liposomes via the layer-by-layer (L-b-L) self-assembly technique [152]. Nanoparticles made of chitosan in association with polyethylene oxide have been used as protein carrier [153]. Moreover, an oral delivery system has been developed by using chitosan and tripolyphosphate. In this system, micro- and nano-particles were entrapped in beads made from chitosan in solution of tripolyphosphate [154].

### 9.7. Antioxidant

The balance between oxidant formation and antioxidant defense in biological systems is important in order to prevent oxidation of biomolecules. The higher oxidation activity in cells, which leads to a higher potential for cell injury, is the cause of cancer, arthritis, neurodegeneration, and aging [155]. Chitin and its derivatives have been reported to have antioxidant properties [156], hence preventive effects on various diseases.

Chitin that was chemically modified to obtain aminoethyl-chitin, showed antioxidant activity against free radicals such as 1,1-diphenyl-2-picrylhydrazyl (DPPH), hydroxyl, superoxide, and peroxyl [157]. In addition, the antioxidant activity of hetero-chitosan has been shown to depend on its deacetylation degree and concentration [158]. It seems that free amino groups play a major role in antioxidant activity of chitinous materials [159]. The proposed mechanism behind this activity is that the free radicals react with NH_2_ groups and these groups absorb hydrogen ion from the solution to produce NH_3_ groups [160].

### 9.8. Antimicrobial Activity

Antifungal and antibacterial activities of chitinous products have been demonstrated in several articles. For example, S. Bautista-Baños *et al.* reported the antifungal activity of chitosan against *Colletotrichum gloeosporioides,* which causes anthracnose in papaya [161]. Chitosan has been reported to possess the antibacterial ability against *E. coli* through cross-linking between chitosan (as cation) and anions over the surface of *E. coli* [162]. It has been shown that culture broth of *Bacillus subtilis* grown in the presence of chitin exhibits antifungal activity on pathogenic *Fusarium oxysporum*, indicating that it can be used as a bio-control agent [163]. In addition, chitosan solution has been shown to inhibit the growth of *Xanthomonas* sp., which is pathogenic to *Euphorbia pulcherrima* [164].

The mechanism of antifungal and antimicrobial activity of chitin and its derivatives has yet to be totally uncovered. In fact there are several proposed mechanisms. One of them is the ability of chitin and its derivatives to activate defense mechanisms of the host organisms [165] such as inducing the accumulation of chitinases and other pathogenesis-related proteins [165]. Another one is leakage in the cell wall of bacteria due to the interaction between positively charged chitosan molecules and the negatively charged surface of the bacteria [166].

### 9.9. Gene Therapy

Being able to deliver a large piece of DNA plays an important role in gene therapy. The carrier must be safe with low immunogenicity. One of the carriers being used—viral vectors—may not be safe enough for targeting cells [167]. Cationic derivatives of chitin are being used to serve this purpose [159]. Galactosylated chitosan has been grafted to dextran to make a liver-specific DNA carrier [168]. Another group has synthesized nanosphere delivery vehicle by salt-induced complex co-acervation of cDNA and chitosan [169]. Chitosan can condense DNA and forms small discrete particles in particular conditions; hence it has many potential applications for gene delivery [170].

### 9.10. Food Technology

Chemical preservatives can be replaced with chitin-based ones. The advantages are two-fold. First, chitinous materials are safer, and second, with their antimicrobial activity; they can protect food products against microbial invasion. Chitosan-based films have been developed to serve this purpose [171]. It has also been applied to improve the preservation of vacuum-packaged processed meat and it could delay the growth of *Entrobacteriaceae*, which are indigenous bacteria in the food products [172]. It seems this antibacterial activity is due to the positive charge of C2 amino group in glucosamine, monomer of chitosan. This positive charge interacts with negatively charged microbial cell membrane and leads to leakage of the intracellular constituents of the microorganisms [173].

### 9.11. Agriculture

Chitin oligosaccharides have been shown to play an important role in defense mechanisms of plants against microbial invasion [174]. They could also promote carrot somatic embryos survival [175]. Chitin fragments can desensitize the perception system of tomato, which can lead to improvement of the defense mechanism in tomato cells [176]. Chitin in the form of lipo-chitin can induce the formation of nodule in soybean root [177]. Rice is another important plant in which chitin fragments have been shown to boost the defense system [178,179].

### 9.12. Bio-Nanotechnology

Bio-nanotechnology, a marriage between biology and nanotechnology, is an emerging field. Through biomimetic approaches and strategies, many micro/nano-systems can be produced. Chitosan has been used to immobilize and pattern biomolecules on microfabricated surfaces [180]. A photolithographic method has been applied to integrate chitosan to micro- and nano-structures, which is an important step toward the fabrication of bioinspired micro-electromechanical systems [181]. Nanoimprinting lithography was used to micro- and nano-pattern chitosan [182]. This micro/nanopatterning enables researchers to use chitosan for bionanotechnology applications such as nanobiodevices. Chitiosan has recently been used in the preparation of graphitic carbon nanocapsules, tungsten carbide and tungsten carbides/graphitic carbon composites [183]. In this system, after preparation of the precursors of chitosan and metal ions, they were carbonized. The system can be regulated by changing the type and/or ratio of the metal [183]. Chitin whiskers have been used to reinforce nanocomposites. It seems this ability mainly depends on chitin whiskers being able to form 3D networks. Any modification by which this network is disrupted results in lowering or loss of this ability [184].

### 9.13. Capacitor and Electrolyte

Electric double layer capacitors are being used as memory back-up tools and energy storage technology. Electrolytes used in these capacitors should have low internal resistance and high capacitance. There are two type of electrolytes; aqueous and nonaqueous. Aqueous electrolytes have high electrical conductivity [185]. KOH and H_2_SO_4_, which are being used to make aqueous electrolytes, are strong base and acid, and thus are hazardous and difficult to handle. It is important to have less toxic or non-toxic aqueous electrolytes that are stable and exhibit good conductivity and low resistance. Yamazaki *et al.* made a gel using a mixture of cellulose, chitin, and H_2_SO_4_, which has all the mentioned features as well as high charge-discharge ability [186].

### 9.14. Heavy Metals and Other Pollutants Removal

The wide usage of heavy metals in industry has caused and continues to cause serious and widespread health problems. It is important to remove these metals from the environment. Several studies have reported the ability of chitin and chitosan to perform this task. For instance, absorbing Cu (II) and Cr (VI) [187], Fe [188], and Pb (II) ions in an aqueous environment [189]. Chitin phosphate could absorb uranium in the presence of sodium carbonate solution [187]. Chitosan-based chelating resins have been developed to absorb mercury [190], Ti (IV), V (V), Mo (VI) [191], W (VI), U (VI) [192], Ag in aquatic environment [193], Cd, Ni, V, Ga, Sc, In, and Th [194]. Chitin and chitosan have been shown to have copper removal capability, which could help to obtain more stable diesel oil [195]. They also have been successfully tested for the adsorption of organic pollutants [196].

### 9.15. Intelligent Materials or Composites

Chitinous materials have been used to create smart or intelligent materials or composites. These systems can respond to environmental changes. They show their functionality with the addition or removal of stimulation. These smart or intelligent materials are ideal in an integrated systems or mixed composite of materials. Shape memory materials are one group of such materials [197]. As is obvious from the name, shape memory materials can remember and regain their original shape after the removal of the stimulus. This phenomenon is due to their being equipped with proper stimulus sensitive molecular switches. Among these shape memory materials, polyurethanes are gaining more attention. This is because of their good shape memory effect at room temperature as well as their low cost. But shape memory polyurethanes cannot bear repeated changes in the shape memory, and retention will decrease by increasing the number of cycles of shape memory; consequently, chitin-based polyurethane shape memory materials have been developed to overcome these problems [198].

### 9.16. Energy Production: An Emerging Application

Insects are widely distributed on earth, comprising 80% of species [199]. Robots with the ability to hunt and digest insects and obtain energy from them can serve humanity by performing missions in dangerous situations. A robot that contains a microbial fuel cell was created to digest chitin and metabolize it by bacteria. This process produces electrons that act as horsepower of the system [200]. The system can take advantage of the wide distribution of arthropods and mollusks because chitin is available in both phyla. Since *Arthropoda* and *Mollusca* rank first and second in species diversity in all animal phyla [201] and are a major source of chitin, this strategy can be highly applicable in both land and marine environments. Chitin has also been utilized by *Clostridium paraputrificum* M-21 to produce hydrogen gas. This gas is considered to be a potential source of alternative energy [202,203]. The advantage of using chitin in this way is that most chitin sources are waste materials, such as shrimp shells; therefore, it is non-food material and there is no need to be concerned about pressure on food supplies.

## 10. Chito-Oligosaccharides and Their Applications

Recent trends in the field of chitin research have focused on oligosaccharides, which are more soluble and have several attractive biological effects. *N*-acetylchitooligosaccharide and chitooligosaccharide (COS) are originated from chitin and chitosan, respectively. Oligomers of chitin and chtitosan can be obtained both chemically and enzymatically [126]. Their degree of polymerization (DP) is usually < 20.

It has been shown that chito-oligosaccharide accelerates the wound healing effects of Poly vinyl alcohol (PVA) if it is used in the early stages of the healing process [204]. It could inhibit the growth of *Actinobacillus actinomycetemcomitans*, indicating that it has antimicrobial activity [205]. *N-*acetylchitooligosaccharide causes an increase in biophotons emission from suspension-cultured rice cells. Biophotons are very weak light emitted from biological processes/systems. All organisms, including plants, constantly produce biophotons as part of their vital activities. Photon emissions are elevated by environmental stresses and disease responses induced by pathogen attack. It has been shown that photon emissions from rice cells elicited by *N*-acetylchitohexaose are closely associated with the ROS-generating system, and are regulated by Ca2+ signaling and protein phosphorylation via phosphatidic acid (PA), an intermediate of phospholipid signaling [206]. Chito-oligosaccharide has shown inhibitory effects on tumor growth and metastasis of lung cancer in mice [207]. *N-*acetylchitohexaose (chitin derivertive) and chitohexaose (chitosan derivertive) can also induce production of interleukins 1 and 2 and, consequently, help to improve the function of macrophages, natural killers, cytotoxic T cells, and polymorphonuclear leukocytes, in defense mechanisms. The anti-metastatic activity of acetylchitohexaose against Lewis lung carcinoma in mice [208] has been demonstrated. There are many reports indicating antitumor activity of chito-oligosaccharides [209]. It has been shown that oligosaccharides that contain *N-*acetylglucosamine play an important role in the interaction between HIV and T-helper during pathogenesis of this virus [210]. *N-*acetylchitooligosaccharide was used as analogue to study lysozyme [211]. Thus, these materials can be used to study protein-carbohydrate interaction and associated enzymatic activities.

## 11. Conclusion and Future Perspectives

After two centuries of research on chitin, this biopolymer now has applications in numerous fields, as described in many review articles [140,212–216]. However, there is still room for further chitin research. Chitin is a potential energy source as well as gene and drug delivery carrier, and in the emerging field of nanobiotechnology. The evolutionary effects of chitin is emerging, but is not yet fully discovered. To take advantage of the mechanical characteristics of chitin, it should be used directly and without chemical modification. To achieve this goal, more research, especially on fundamental aspects, needs to be done.

Much attention has being paid to chitinase research [75], but its substrate seems to have been neglected or, at least, underestimated. If we are going to use the huge amount of chitin produced annually in nature, chitin research needs an increase in funding to develop equipment to harvest chitin from its diverse sources, improve the purity of obtained chitin, and produce novel materials with new applications from this environmentally friendly biopolymer.

Despite the multiple potential applications of chitin, we believe that the most promising in the future are (i) applications in nanobiotechnology, which involves drug and gene delivery and scaffold for tissue engineering, and (ii) applications of chito-oligosaccharides in medicine and agriculture.
